# Mechanistic insight into the norepinephrine-induced fibrosis in systemic sclerosis

**DOI:** 10.1038/srep34012

**Published:** 2016-09-21

**Authors:** Akihito Uehara, Sei-ichiro Motegi, Kazuya Yamada, Akihiko Uchiyama, Buddhini Perera, Sayaka Toki, Sachiko Ogino, Yoko Yokoyama, Yuko Takeuchi, Osamu Ishikawa

**Affiliations:** 1 Department of Dermatology, Gunma University Graduate School of Medicine, Japan

## Abstract

Raynaud’s phenomenon is frequently observed in systemic sclerosis (SSc) patients, and cold- or stress-induced norepinephrine (NE) has been speculated to be associated with vasoconstriction. Objective was to elucidate the role of NE in fibrosis in SSc. IL-6 is a potent stimulator of collagen production in fibroblasts. NE enhanced IL-6 production and proliferation more significantly in SSc fibroblasts than in normal fibroblasts. Furthermore, the production of IL-6 and phosphorylation of p38 in SSc fibroblasts was enhanced by adrenergic receptor (AR)β agonist, isoproterenol, but not ARα agonist, oxymetazoline. ARβ blocker, propranolol, inhibited NE-induced IL-6 production and phosphorylation of p38 in SSc fibroblasts. NE-induced IL-6 was significantly inhibited by p38 inhibitor, SB203580, suggesting that NE-induced phosphorylation of p38 via ARβ enhances IL-6 production in SSc fibroblasts. NE-induced phosphorylation of ERK1/2 via ARα inhibited IL-6 production in SSc fibroblasts. Combined treatment with NE and endothelin-1 resulted in an additive increase in IL-6 production in SSc fibroblasts. NE-induced IL-6/IL-6 receptor trans-signaling increased the production of collagen type I in SSc fibroblasts, and both propranolol and SB203580 inhibited NE-induced collagen production. These results suggest that cold exposure and/or emotional stress-induced NE might contribute to the skin fibrosis via potentiation of IL-6 production from fibroblasts in SSc.

Norepinephrine (NE) is primarily released from the postganglionic neurons of the sympathetic nervous system as a neurotransmitter. NE is also synthesized in the locus coeruleus and adrenal medulla. It is well known that emotional stress and cold stimulation increase the systemic and/or local NE levels^1^,^2^,^3^. NE binds to the adrenergic receptor (AR) in multiple organs, including the heart, lungs, brain and skin. In the skin, ARβ2 is primarily expressed on the surface of keratinocytes, dermal fibroblasts and melanocytes^4^,^5^,^6^. Activation of ARβ2 signaling impairs re-epithelialization, resulting in delayed wound healing in both human and murine skin^7^,^8^. In *in vitro* studies, activation of ARβ2 by isoproterenol enhances migration and alters both the actin cytoskeleton and focal adhesion distribution in dermal fibroblasts, suggesting that NE:AR signaling regulates the function of dermal fibroblasts^7^,^8^,^9^.

Systemic sclerosis (SSc) is a connective tissue disorder characterized by the development of fibrosis in the skin and internal organs as well as microvascular dysfunction. Raynaud’s phenomenon is commonly observed in patients with SSc and characterized by the presence of episodic vasospasms and ischemia of the extremities in response to cold or emotional stress. It has also been speculated that cold- or stress-induced NE stimulates ARα on pericytes and/or vascular smooth muscle cells, thus resulting in vasoconstriction^10^,^11^. In addition, SSc patients treated with the ARα_2c_ antagonist exhibit improvements in the symptoms of Raynaud’s phenomenon induced by cold stimulation^12^, suggesting that NE is involved in the pathogenesis of vasculopathy in SSc. However, the roles of NE in the development of skin fibrosis associated with SSc are not well investigated.

IL-6 is a pleiotropic multifunctional cytokine produced by various cells, such as lymphocytes, monocytes and fibroblasts^13^. IL-6 has various immunological functions, for example, it induces B cell differentiation to produce immunoglobulin, stimulates Th17 differentiation in the presence of transforming growth factor (TGF)-β and inhibits the induction of TGFβ-induced regulatory T cells^14^,^15^. In addition, IL-6 is considered to be involved in the pathogenesis of several autoimmune diseases, including SSc and rheumatoid arthritis^16^. With respect to IL-6 and SSc, many *in vivo* and *in vitro* studies have shown that IL-6 plays an important role in the pathogenesis of fibrosis in SSc. For example, the serum IL-6 levels are significantly elevated in SSc patients of early stage^17^,^18^ and correlate with the total skin thickness score in persons with this disease^19^. Moreover, a prominent expression of IL-6 is observed in dermal fibroblasts, mononuclear cells and endothelial cells in the patient’s skin of early stage of diffuse cutaneous type (dc)SSc^18^,^20^, suggesting that dermal fibroblasts are an important source of IL-6 in affected skin lesions. In *in vitro* studies, skin dermal fibroblasts derived from SSc patients have been found to produce high levels of IL-6, and the complex of IL-6 and soluble IL-6 receptor (sIL-6R) has been shown to stimulate SSc fibroblasts via gp130 to differentiate and proliferate, resulting in collagen overproduction and fibrosis^18^,^21^,^22^,^23^. In addition, several case studies have reported softening of the skin in SSc patients after the treatment with an anti-IL-6 receptor antibody (tocilizumab), supporting the essential role of IL-6 in the pathogenesis of skin fibrosis associated with SSc^24^. Recently, phase II clinical trial on tocilizumab in SSc patients revealed that the reduction of skin sclerosis in tocilizumab group tended to be greater than those in placebo group^25^. It has also been reported that IL-1α, platelet-derived growth factor (PDGF), tumor necrosis factor-α (TNF-α) and CD154/CD40 interactions induce IL-6 production in fibroblasts^22^,^26^,^27^,^28^. However, the precise mechanisms underlying the onset of IL-6-induced fibrosis in the setting of SSc and whether NE stimulation enhances IL-6 production in SSc fibroblasts remain unclear. In addition, the relationship between NE and endothelin-1 (ET-1), which is associated with skin fibrosis in SSc patients, in the pathogenesis of skin sclerosis of SSc is unknown. In this study, we examined the mechanisms of NE-induced IL-6 production in SSc fibroblasts and aimed to clarify the roles of NE in the pathogenesis of fibrosis in SSc.

## Results

### NE-induced IL-6 production was significantly higher in the SSc fibroblasts than in the normal fibroblasts

**Figure 1:**
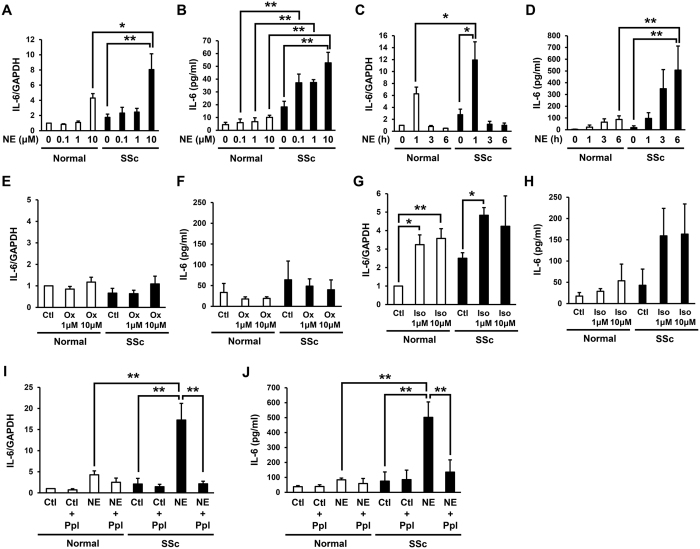
NE-induced IL-6 production via ARβ and NE-induced proliferation in SSc fibroblasts were significantly higher than those in normal fibroblasts. (**A,B**) Effects of NE on IL-6 mRNA (**A**) and IL-6 protein (**B**) secreted into the media from normal and SSc fibroblasts. Normal and SSc fibroblasts were incubated during 1 hour with indicated concentration of NE. (C, D) Effects of NE on IL-6 mRNA (**C**) and IL-6 protein (**D**) secreted into the media from normal and SSc fibroblasts. Normal and SSc fibroblasts were incubated with 10 μM NE for indicated time. (E, F) IL-6 mRNA (**E**) and secreted protein (**F**) into the media in normal and SSc fibroblasts treated with oxymetazoline (Ox: ARα agonist) for 1 hour. (**G,H**) IL-6 mRNA (**G**) and secreted protein (**H**) into the media in normal and SSc fibroblasts treated with isoproterenol (Iso: ARβ agonist) for 1 hour. (**I,J**) IL-6 mRNA (I) and secreted protein (J) into the media in normal and SSc fibroblasts treated with NE (10 μM) and/or ARβ inhibitor, propranolol (Ppl) for 1 hour. n = 3-6 samples. mRNA levels in normal fibroblasts without treatments were assigned a value of 1. All values represent mean ± SEM. **P < 0.01, *P < 0.05.

In order to assess the effects of NE on IL-6 production from normal and SSc dermal fibroblasts, the secreted protein and mRNA levels of IL-6 in normal and SSc fibroblasts treated with NE were examined. The mRNA and secreted protein levels of IL-6 in the normal and SSc fibroblasts were increased by NE stimulation for one hour in a dose-dependent manner (Fig. 1A,B). Furthermore, the IL-6 mRNA and secreted protein levels in the SSc fibroblasts treated with NE were significantly higher than those observed in the normal fibroblasts treated with NE (Fig. 1A,B). Next, we analyzed the IL-6 mRNA and secreted protein levels in the normal and SSc fibroblasts treated with 10 μM of NE for the indicated time. The IL-6 mRNA levels in the normal and SSc fibroblasts increased after NE stimulation, peaking at one hour of stimulation, and then decreased to the basal values at three hours after stimulation (Fig. 1C). In addition, the IL-6 mRNA levels in the SSc fibroblasts treated with 10 μM of NE for one hour were significantly higher than those in the normal fibroblasts (Fig. 1C). The secreted protein levels of IL-6 from the normal and SSc fibroblasts were increased by NE treatment in a time-dependent manner (Fig. 1D), and the IL-6 secreted protein levels from the SSc fibroblasts treated with 10 μM of NE for six hours were significantly higher than those in the normal fibroblasts (Fig. 1D). These results suggest that NE enhances IL-6 production in normal and SSc fibroblasts in a dose- and time-dependent manner and that the NE-induced IL-6 production is significantly higher in SSc fibroblasts than in normal fibroblasts.

### NE-induced IL-6 production in both the normal and SSc fibroblasts was mediated primarily via ARβ

NE binds both ARα and ARβ to activate the downstream signaling. However, NE has different mechanisms of activation for ARα and ARβ, depending on the specific G protein subunits activated^29^,^30^. In order to examine whether the NE-induced IL-6 expression is conducted via ARα or ARβ, we assessed the IL-6 mRNA and secreted protein levels in normal and SSc fibroblasts treated with oxymetazoline (ARα agonist) or isoproterenol (ARβ agonist) for one hour. While oxymetazoline did not affect the IL-6 mRNA and secreted protein levels in the normal or SSc fibroblasts (Fig. 1E,F), isoproterenol elevated the IL-6 mRNA and secreted protein levels in both the normal and SSc fibroblasts (Fig. 1G,H). Next, we examined the effects of the ARβ inhibitor, propranolol, on NE-induced IL-6 production in normal and SSc fibroblasts. Consequently, propranolol significantly inhibited the NE-induced IL-6 mRNA and secreted protein levels in the SSc fibroblasts (Fig. 1IJ). These results suggest that the NE-induced IL-6 production in normal and SSc fibroblasts is mediated primarily via ARβ.

### Expression of ARα and ARβ in the normal and SSc fibroblasts

Next, we explored the expression of AR in normal and SSc fibroblasts treated with or without NE. Consequently, NE did not affect the ARα1B, ARα1D or ARβ1 expression levels in the normal or SSc fibroblasts (Supplemental Figure S1A–C). There was a tendency for the ARβ2 expression to be higher in the normal and SSc fibroblasts treated with NE than in those treated without NE, however, there were no significant differences (Supplemental Figure S1D). In addition, there was a tendency for the ARα1 expression to be higher in SSc fibroblasts than in normal fibroblasts (Supplemental Figure S1A,B). However, there were no significant differences in the surface protein expression levels of ARα1 between the normal and SSc fibroblasts by immunofluorescence staining (Supplemental Figure S1E).

### NE-induced phosphorylation of p38 via ARβ enhanced the IL-6 production in the SSc fibroblasts

It has been reported that stimulation of ARβ enhances the phosphorylation of p38 in cardiac fibroblasts^31^. In addition, p38 is known to be involved in the production of IL-6 in synovial and gingival fibroblasts^32^,^33^. These findings suggest that p38 signaling is associated with NE-induced IL-6 production in human dermal fibroblasts. In order to assess this hypothesis, we examined the phosphorylation of p38 in normal and SSc fibroblasts treated with NE, oxymetazoline or isoproterenol.

**Figure 2:**
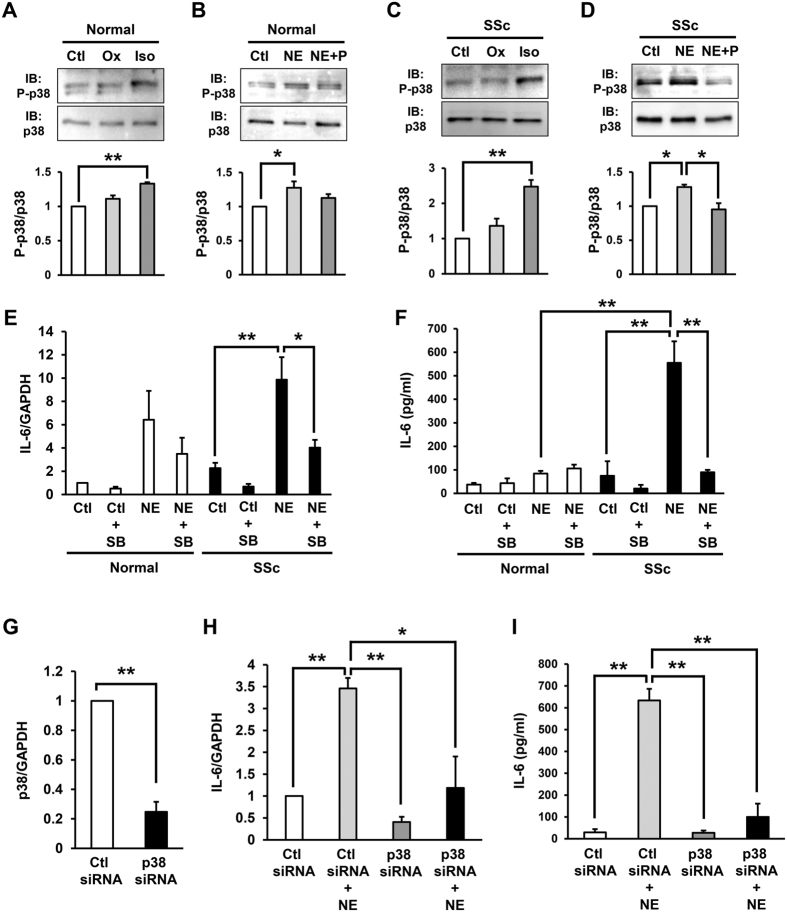
NE-induced phosphorylation of p38 via ARβ enhanced IL-6 production in SSc fibroblasts. (**A,C**) Phosphorylation of p38 in normal (**A**) and SSc (**C**) fibroblasts treated with oxymetazoline (Ox) or isoproterenol (Iso) by immunoblotting. (**B,D**) Phosphorylation of p38 in normal (**B**) and SSc (**D**) fibroblasts treated with NE and/or ARβ inhibitor, propranolol (P) by immunoblotting. Quantification of relative phosphorylation levels of p38 was accomplished via densitometry using ImageJ. The level of p38 phosphorylation in normal or SSc fibroblasts without treatment was assigned a value of 1. n = 3 samples. Cropped blots were used, full-length blots are in Supplementary Figure S3. All gels have been run under the same experimental conditions. (**E,F**) IL-6 mRNA (**E**) and secreted protein (**F**) into the media in normal and SSc fibroblasts treated with NE (10 μM) and/or p38 inhibitor, SB203580 (SB) for 1 hour. n = 3 samples. mRNA levels in normal fibroblasts without treatments were assigned a value of 1. (**G**) p38 mRNA in SSc fibroblasts transfected with control or p38 siRNA. (**H,I**) IL-6 mRNA (**H**) and secreted protein (**I**) into the media in SSc fibroblasts transfected with control or p38 siRNA treated with or without NE. mRNA levels in control siRNA transfected SSc fibroblasts without treatments were assigned a value of 1. n = 3 samples. All values represent mean ± SEM. **P < 0.01, *P < 0.05.

The phosphorylation of p38 in the normal and SSc fibroblasts was significantly enhanced by the ARβ agonist, isoproterenol, but not the ARα agonist, oxymetazoline (Fig. 2A,C). In addition, the phosphorylation of p38 in the normal and SSc fibroblasts was significantly enhanced by NE, and the NE-induced p38 phosphorylation was inhibited by the ARβ inhibitor, propranolol (Fig. 2B,D). These results suggest that NE-induced p38 phosphorylation is primarily mediated via ARβ. Furthermore, the NE-induced IL-6 mRNA and secreted protein levels in the SSc fibroblasts was significantly inhibited by the p38 inhibitor, SB203580 (Fig. 2E,F). Furthermore, to confirm the role of p38 in NE-induced IL-6 production, the effects of siRNA depletion of p38 in SSc fibroblasts were analysed. The expression of p38 mRNA in SSc fibroblasts treated with siRNA of p38 was reduced by approximately 80%, compared with that in the fibroblasts treated with control siRNA (Fig. 2G). siRNA depletion of p38 significantly inhibited NE-induced IL-6 mRNA and secreted protein levels in SSc fibroblasts (Fig. 2H,I). These results suggest that the NE-induced phosphorylation of p38 via ARβ enhances IL-6 production in SSc fibroblasts.

### NE-induced phosphorylation of ERK1/2 via ARα inhibited the IL-6 production in the SSc fibroblasts

**Figure 3:**
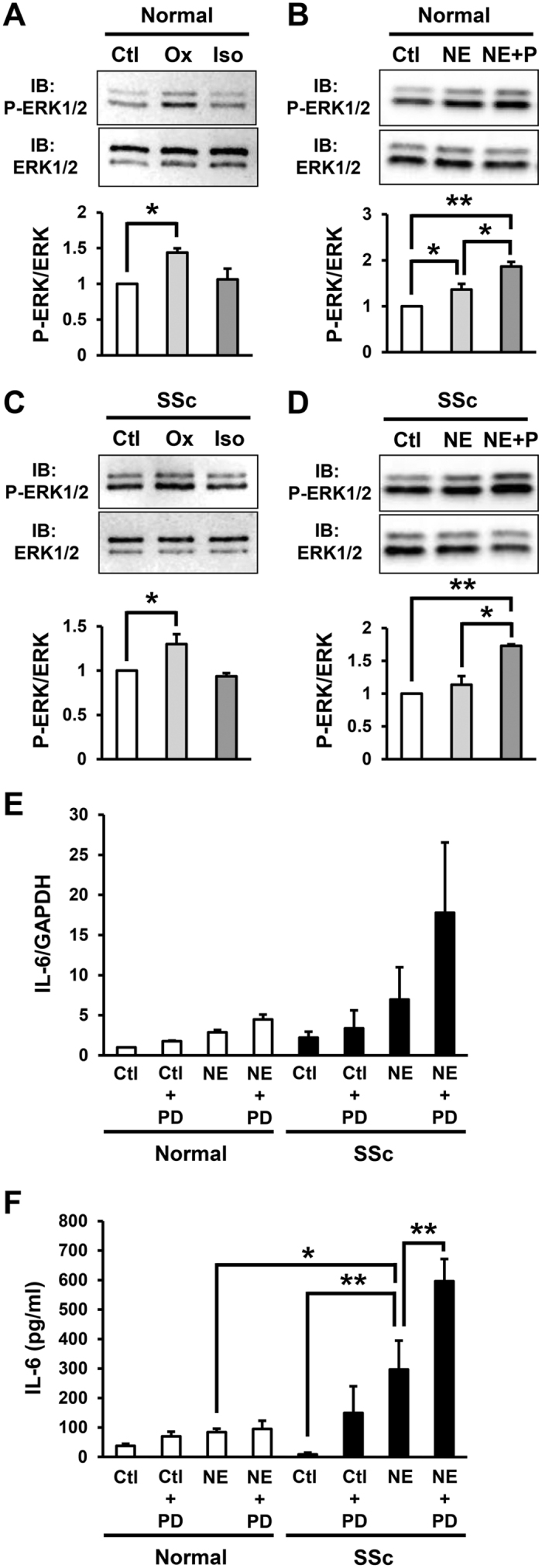
NE-induced phosphorylation of ERK1/2 via ARα inhibited IL-6 production in SSc fibroblasts. (**A,C**) Phosphorylation of ERK1/2 in normal (**A**) and SSc (**C**) fibroblasts treated with oxymetazoline (Ox) or isoproterenol (Iso) by immunoblotting. (**B,D**) Phosphorylation of ERK1/2 in normal (**B**) and SSc (**D**) fibroblasts treated with NE and/or ARβ inhibitor, propranolol (P) by immunoblotting. Quantification of relative phosphorylation levels of ERK1/2 was accomplished via densitometry using ImageJ. The level of ERK1/2 phosphorylation in normal or SSc fibroblasts without treatment was assigned a value of 1. n = 3 samples. Cropped blots were used, full-length blots are in Supplementary Figure S4. All gels have been run under the same experimental conditions. (**E,F**) IL-6 mRNA (**E**) and secreted protein (**F**) into the media in normal and SSc fibroblasts treated with NE and/or MEK/ERK inhibitor, PD98059 (PD) for 1 hour. n = 3 samples. mRNA levels in normal fibroblasts without treatments were assigned a value of 1. All values represent mean ± SEM. **P < 0.01, *P < 0.05.

Next, we examined the role of ERK1/2 in the NE-induced IL-6 production in fibroblasts. ERK1/2 is known to be involved in the downstream pathway of AR^29^,^30^. In this study, the phosphorylation of ERK1/2 in the normal and SSc fibroblasts was significantly enhanced by the ARα agonist, oxymetazoline, but not the ARβ agonist, isoproterenol (Fig. 3A,C). Moreover, the phosphorylation of ERK1/2 in the normal and SSc fibroblasts was enhanced by NE, while the NE-induced ERK1/2 phosphorylation was enhanced by the ARβ inhibitor, propranolol (Fig. 3B,D). These results suggest that NE-induced ERK1/2 phosphorylation is primarily mediated via ARα. Furthermore, the NE-induced IL-6 mRNA and secreted protein levels in the normal and SSc fibroblasts was enhanced by the MEK/ERK inhibitor, PD98059 (Fig. 3E,F), thus suggesting that the NE-induced phosphorylation of ERK1/2 via ARα inhibits IL-6 production in SSc fibroblasts.

### Combined treatment with NE and ET-1 resulted in an additive increase in the production of IL-6 in the SSc fibroblasts

**Figure 4:**
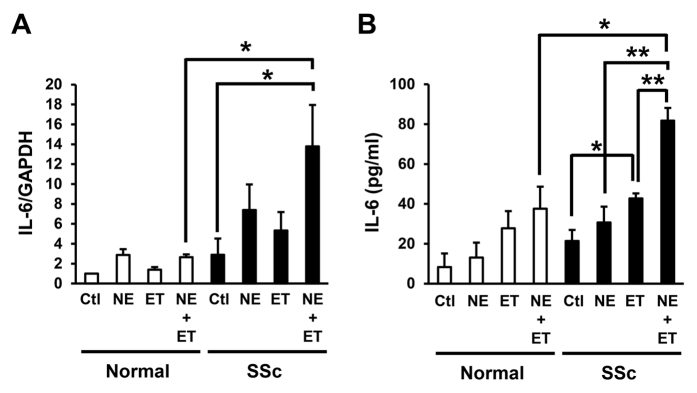
Combined treatment with NE and ET-1 resulted in an additive increase in the production of IL-6 in SSc fibroblasts. IL-6 mRNA (**A**) and secreted protein (**B**) into the media in normal and SSc fibroblasts treated with NE (10 μM) and/or ET-1 (1 μM) for 1 hour. n = 3 samples. mRNA levels in normal fibroblasts without treatments were assigned a value of 1. All values represent mean ± SEM. **P < 0.01, *P < 0.05.

ET-1 has reported to be been involved in the pathogenesis of fibrosis in various organs, including the skin, lungs and heart^34^. ET-1 induces the production of collagen type I and III and fibronectin via ET receptors A and B on fibroblasts^35^. In addition, the plasma ET-1 levels are higher in patients with SSc than in normal subjects^36^, suggesting that ET-1 is associated with the pathogenesis of fibrosis in the setting of SSc. Therefore, we next examined the effects of ET-1 on the NE-induced IL-6 production in the normal and SSc fibroblasts. As a result, the IL-6 mRNA and protein levels in the SSc fibroblasts were enhanced by treatment with ET-1 for one hour (Fig. 4A,B). In addition, the IL-6 mRNA and protein levels were significantly enhanced by combined treatment with NE and ET-1 compared to that achieved with treatment with NE or ET-1 alone (Fig. 4A,B), suggesting that the NE-induced IL-6 production in SSc fibroblasts is additively enhanced by simultaneous treatment with ET-1.

### NE-induced IL-6/IL-6 receptor trans-signaling increased the production of collagen type I in SSc fibroblasts

**Figure 5:**
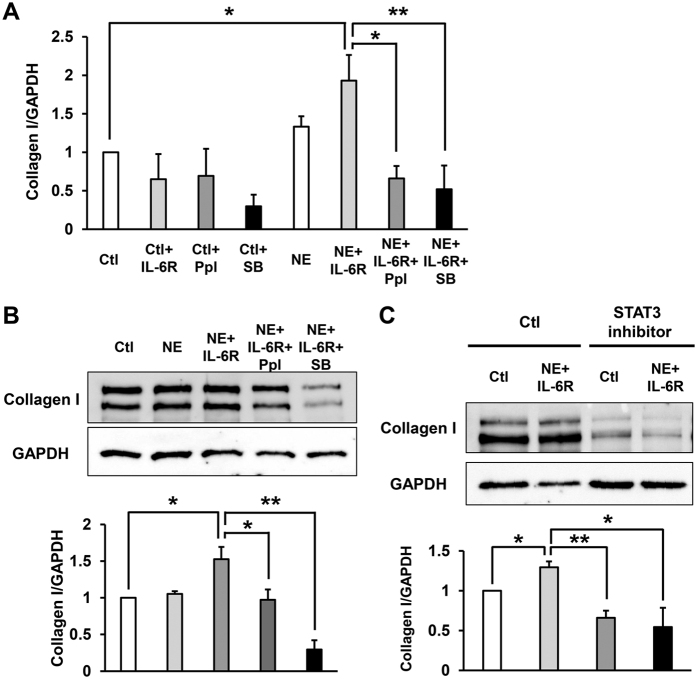
Combined treatment with NE and IL-6 receptor resulted in an increase in the production of collagen type I in SSc fibroblasts. (**A**) Collagen type I mRNA in SSc fibroblasts treated with NE, sIL-6 receptor (IL-6R), propranolol (Ppl) or p38 inhibitor, SB203580 (SB) for 48 hours. n = 3 samples. mRNA levels in fibroblasts without treatments were assigned a value of 1. (**B**) Collagen type I in SSc fibroblasts treated with NE, sIL-6 receptor (IL-6R), propranolol or SB203580 for 48 hours by immunoblotting. (**C**) Collagen type I in SSc fibroblasts treated with NE, sIL-6 receptor (IL-6R) or STAT3 inhibitor (S31-201) for 48 hours by immunoblotting. Quantification of relative levels of collagen type I was accomplished via densitometry using ImageJ (normalized to GAPDH protein levels). The level of collagen type I in fibroblasts without treatment was assigned a value of 1. n = 3 samples. Cropped blots were used, full-length blots are in Supplementary Figure S5. All gels have been run under the same experimental conditions. All values represent mean ± SEM. **P < 0.01, *P < 0.05.

It has been reported that IL-6 complexes with a sIL-6R to associate with gp130 on the cell surface and initiate intracellular signaling, which is termed “trans-signaling”^18^,^37^,^38^. In addition, increased levels of serum IL-6 secreted from many cells interact with sIL-6R, enhancing activation of endothelial cells, expression of adhesion molecules and apoptosis, which seem to occur early in the development of SSc^38^. Therefore, we finally examined the role of NE-induced IL-6 and sIL-6R trans-signaling in fibrosis in SSc fibroblasts. Incubation of SSc fibroblasts with NE alone had no significant effect of both mRNA and protein levels of collagen production (Fig. 5A,B). However, incubation of SSc fibroblasts with NE and sIL-6R together significantly increased collagen type I production (Fig. 5A,B). In addition, the ARβ inhibitor, propranolol, and the p38 inhibitor, SB203580 significantly inhibited the NE and IL-6R-induced collagen type I production in the SSc fibroblasts (Fig. 5A,B). Furthermore, we examined the effect of STAT3 inhibitor, S31-201 on the NE-induced IL-6/IL-6R trans-signaling, and identified that STAT3 inhibitor significantly inhibited the NE and IL-6R-induced collagen type I production in the SSc fibroblasts (Fig. 5C). These results suggest that the NE-induced IL-6/IL-6R trans-signaling might enhance skin fibrosis in SSc, and ARβ inhibitor, propranolol, the p38 inhibitor, SB203580 and STAT3 inhibitor, S31-201 may suppress NE-induced skin fibrosis.

### NE-induced proliferation was significantly higher in the SSc fibroblasts than in the normal fibroblasts

We then assessed the effects of NE on proliferation in the normal and SSc dermal fibroblasts. The degree of proliferation in the SSc fibroblasts was increased by treatment with NE (Supplemental Figure S2). In addition, the rate of proliferation was significantly higher in the SSc fibroblasts treated with NE than in the normal fibroblasts (Supplemental Figure S2). These results suggest that the NE-induced proliferation is significantly higher in SSc fibroblasts than in normal fibroblasts.

## Discussion

There have been a number of reports suggesting the possible involvement of stress and/or the stress-adaptation system in the pathogenesis of rheumatoid arthritis and systemic lupus erythematosus (SLE)^39^,^40^. It has also been reported that the serum NE levels are significantly higher in SSc patients than in normal individuals^1^. In addition, the mental stress-induced serum NE levels are also higher in SSc patients than in normal individuals based on mental calculation stress tests, suggesting that SSc patients may have an impaired function of the neuro-endocrine-immune system due to stress^1^. Previous findings showed that serum NE levels in normal individuals were increased after cold exposure^2^, and serum NE levels increased after repeated cold-water immersions^3^, further indicate that emotional stress and cold exposure increase the systemic and/or local NE levels in SSc patients.

In this study, we demonstrated that NE stimulation increases the IL-6 expression in dermal fibroblasts in both normal and SSc patients and that NE enhances IL-6 production in SSc fibroblasts more so than in normal fibroblasts. Since the peripheral tissues of the extremities, including the fingers and toes, are likely to be exposed to cold stimulation, cold stimulation-induced NE may increase the IL-6 concentrations locally, resulting in peripheral skin sclerosis in SSc patients. This may also explain why the onset of skin fibrosis initially starts from the acral parts of the extremities. Furthermore, our results suggest that avoiding cold exposure or emotional stress may suppress both fibrosis and Raynaud’s phenomenon in SSc.

**Figure 6:**
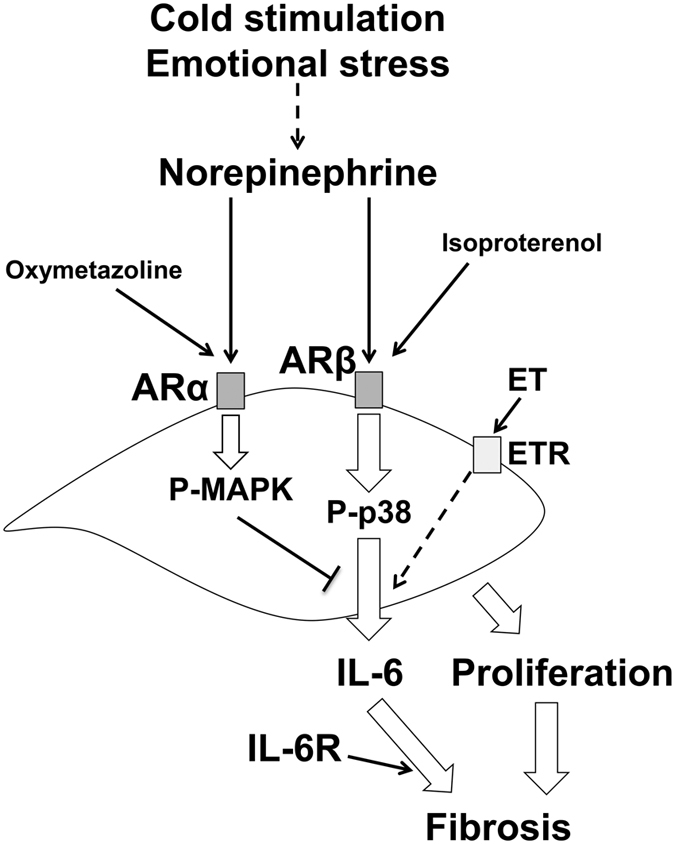
Model for the regulation of IL-6 production by NE in SSc fibroblasts. Emotional stress and/or cold exposure might increase systemic and/or local NE levels. Production of IL-6 in SSc fibroblasts was enhanced by both NE and ARβ agonist, isoproterenol, but not ARα agonist, oxymetazoline, suggesting that NE-induced IL-6 production is primarily mediated via ARβ. NE binds to and activates the downstream signaling of ARβ, thus leading to the phosphorylation of p38. This NE-induced activation of p38 signaling may consequently enhance IL-6 production in SSc fibroblasts. In contrast, the NE-induced phosphorylation of ERK1/2 via ARα subsequently results in the inhibition of IL-6 production in SSc fibroblasts. NE-induced IL-6 production and proliferation of fibroblasts might lead to skin fibrosis in SSc. ET: endothelin. ETR: endothelin receptor.

Moreover, we elucidated the mechanisms underlying the NE-induced IL-6 production in SSc fibroblasts and subsequently propose a model for the regulation of IL-6 production by NE in SSc fibroblasts (Fig. 6). In this study, the production of IL-6 in the SSc fibroblasts was enhanced by both NE and the ARβ agonist, isoproterenol, but not the ARα agonist, oxymetazoline, suggesting that the NE-induced IL-6 production is primarily mediated via ARβ. NE binds to and activates the downstream signaling of ARβ, thus leading to the phosphorylation of p38. This NE-induced activation of p38 signaling may consequently enhance IL-6 production in SSc fibroblasts. In contrast, the NE-induced phosphorylation of ERK1/2 via ARα subsequently results in the inhibition of IL-6 production in SSc fibroblasts. It is thought that IL-6 is able to bind to the soluble IL-6 receptor secreted from B cells, T cells and monocytes and thus form a complex with gp130 on fibroblasts that enhances collagen production^18^,^20^,^21^,^22^,^23^. In addition, it has been recognized that STAT3 is important in the IL-6/IL-6R trans-signaling in fibroblasts^41^. We demonstrated that NE-induced IL-6/IL-6 receptor trans-signaling via STAT3 activation increased collagen type I production in SSc fibroblasts, suggesting that NE induces IL-6 secretion from dermal fibroblasts and has both paracrine and autocrine effects on various cells, including dermal fibroblasts, T cells and B cells, that stimulate skin fibrosis in the setting of SSc. The NE-induced proliferation of fibroblasts may also lead to skin fibrosis in SSc patients. Further elucidating the regulatory mechanisms mediated by NE:AR signaling may provide new insight into the pathogenesis of skin sclerosis associated with SSc.

It has been known that ARβ stimulation increases cyclic AMP (cAMP), leading to the activation of protein kinase A (PKA)^42^. Wang *et al*. reported that prostaglandin E_2_ enhanced IL-6 production via cAMP/PKA pathway in human chondrocytes, suggesting that NE-induced IL-6 might be mediated via cAMP/PKA pathway^43^. However, we have not examined the involvement of cAMP/PKA pathway in the present study, therefore, further examinations are needed.

In addition, the NE-enhanced IL-6 production and proliferation are greater in SSc fibroblasts than in normal fibroblasts. We examined AR expression in normal and SSc fibroblasts and found that there were no significant differences between normal and SSc fibroblasts with or without NE treatment. These results suggest that the AR expression is not associated with the mechanisms underlying the higher NE-induced IL-6 production observed in SSc fibroblasts than in normal fibroblasts. The mechanisms underlying the hyperreactivity of IL-6 production in SSc fibroblasts treated with NE are unknown, and further studies are thus required to clarify these processes.

In this study, we found that the NE-induced IL-6 production in SSc fibroblasts is additively enhanced by treatment with ET-1, suggesting that NE:ARβ signaling and ET-1:ET receptor signaling additively enhance IL-6 production in these cells. Similar to our results, it has been previously reported that ET-1, acting via the ET receptor A and PKC activation, is an essential co-factor for the ARβ-induced proliferation of human cardiac fibroblasts^44^. The current findings provide new insight into the relationship between NE:AR signaling and ET-1:ET receptor signaling in SSc fibroblasts, although further investigations are also required to elucidate the precise mechanisms underlying the additive effects of ET-1 on the NE-induced IL-6 production in SSc fibroblasts.

In conclusion, we herein demonstrated that NE enhances IL-6 production in SSc fibroblasts via ARβ, subsequently enhances collagen production in SSc fibroblasts. p38 activation is involved in this mechanism, and both cold exposure and emotional stress may initiate this process. We for the first time elucidated the possible link between peripheral circulation disturbance and tissue fibrosis via through NE stimulation, which will provide us with novel therapeutic strategy for SSc. The avoidance of cold exposure or emotional stress may attribute to the suppression of fibrosis as well as Raynaud’s phenomenon in SSc. In addition, we speculate that cold exposure-induced NE may explain the reason why the vasculopathy and skin fibrosis initially start from acral parts of extremities in SSc patients. In the current study, the ARβ blocker, propranolol, the p38 inhibitor, SB203580, and STAT3 inhibitor, S31-201 inhibited the NE-induced IL-6 production and fibrosis in SSc fibroblasts *in vitro*, suggesting that ARβ blocker, p38 and/or STAT3 inhibitor therapy can be an alternative treatment for skin sclerosis in patients with SSc^45^.

## Methods

### Reagents

We used (±)-norepinephrine (+)-bitartrate salt (Sigma-Aldrich, St Louis, MO), isoproterenol hydrochloride (Millipore, Billerica, MA), propranolol hydrochloride (Wako, Osaka, Japan), oxymetazoline hydrochloride (Wako), p38 inhibitor, SB203580 (Wako), MEK/ERK inhibitor, PD98059 (Millipore), STAT3 inhibitor, S31-201 (Sigma-Aldrich), recombinant human TGFβ1 (rTGFβ) (R&D systems, Minneapolis, MN), ET-1 (Sigma-Aldrich) and soluble IL-6 receptor (sIL-6R) (Peprotech, Rocky Hill, NJ).

### Patients

We obtained human dermal fibroblasts by skin biopsies of affected dorsal forearm areas from 6 dcSSc patients and age, race and gender matched 6 healthy volunteers. All SSc patients were <2 years of skin thickening and fulfilled the criteria for SSc proposed by American College of Rheumatology^46^. The study was approved by the research ethics committee/human genome, gene analysis research ethics committee of Gunma University (#185). All patients and volunteers provided written informed consent before participation. This study was conducted according to the Declaration of Helsinki principles.

### Cell cultures

Human dermal fibroblasts were cultured in Dulbecco’s modified Eagle’s medium (DMEM) containing 100 units/ml penicillin, 100μg/ml streptomycin and 10% fetal calf serum (FCS) and used before passage 10. Human dermal fibroblasts were incubated in DMEM with or without NE, oxymetazoline or isoproterenol at indicated concentration for indicated time. To examine the effect of the ARβ inhibitor, propranolol, the MEK/ERK inhibitor, PD98059, p38 inhibitor, SB203580 or STAT3 inhibitor, S31-201 on NE-induced IL-6 production, cells were pretreated with 1 μM propranolol, 50 μM PD98059, 10 μM SB203580 or 100 μM S31-201 or DMSO (vehicle control) for 30 minutes, and then stimulated with NE (10 μM) and/or sIL-6 receptor (IL-6R: 100 ng/ml). The concentration of NE (0-10 μM), propranolol (1 μM), PD98059 (50 μM), SB203580 (10 μM) and S31-201 (100 μM) was referred from previously reported manuscripts^31^,^47^,^48^,^49^,^50^. To inhibit the expression of p38, fibroblasts were transfected with 100 nM p38 siRNA (Cell Signaling Technology, Danvers, MA). After 48 hours, mRNA levels were assessed by quantitative RT-PCR. Fibroblasts were treated with NE (10 μM) for 1 hour at 48 hours after transfection of siRNA.

### Real-time polymerase chain reaction (PCR) analysis

Total RNA was isolated by RNeasy Mini Kits (Qiagen, Valencia, CA) and was subjected to reverse transcription with the use of a Superscript III First-Strand Synthesis System for reverse transcription (RT)-PCR (Invitrogen) according to previously described protocols^49^,^51^. Quantitative RT-PCR was performed using the Taqman system (Applied Biosystems, Foster City, CA) using 7300 Real Time PCR systems (Applied Biosystems) according to the manufacturer’s instructions. Taqman probes and primers for IL-6, ARα1B, ARα1D, ARβ1, ARβ2, and glyceraldehyde-3-phosphate dehydrogenase (GAPDH) were purchased from Applied Biosystems. As an internal control, levels of GAPDH were quantified in parallel with target genes. Normalization and fold changes were calculated using the comparative Ct method.

### Measurement of IL-6 concentration

The specific enzyme-linked immunosorbent assay (ELISA) kit was used for measuring IL-6 (R&D systems) in supernatants of the culture according to the manufacture’s protocol.

### Western blot assay

Western blotting and analyses were performed according to previously described protocols^49^. Human dermal fibroblasts were incubated in normal medium with or without oxymetazoline (1 μM) or isoproterenol (1 μM) for 1 hour. Cells were pretreated with 1 μM propranolol, 10 μM SB203580, 100 μM S31-201 or DMSO (vehicle control) for 30 minutes, and then stimulated with NE (10 μM) and/or sIL-6 receptor (IL-6R: 100 ng/ml). After washing with ice-cold PBS, cells were disrupted in lysis buffer (20 mM Tris-HCl pH 7.6, 140 mM NaCl, 1% Nonidet P-40) containing 1 mM phenylmethylsulfonyluoride, aprotinin (10 mg/ml) and 1 mM sodium vanadate. Lysates were centrifuged at 10,000 × g for 15 min at 4 °C and the resulting supernatants were subjected to SDS-PAGE, followed by immunoblot analysis using anti-phospho-p38 MAPK (Thr180/Tyr182) Ab, anti-p38 MAPK Ab, anti-phospho-p44/42 MAPK (Erk1/2) (Thr202/Tyr204) Ab, anti-44/42 MAPK (Erk1/2) Ab (Cell Signaling, Danvers, MA), anti-collagen type I Ab (SouthernBiotech, Birmingham, AL) and anti-GAPDH Ab (Santa Cruz Biotechnology, Dallas, Texas). Anti-rabbit HRP-conjugated secondary antibodies (Jackson ImmunoResearch, West Grove, PA) were used with ECL (Thermo Scientific, Rockford, IL) to image immunoblots. Quantification of protein band intensity was performed using ImageJ software version 1.46r (NIH) as previously reported^49^.

### Proliferation assays

Human dermal fibroblasts (5 × 10^5^ cells per well) were plated in a 6 cm^2^ dish and starved in serum free medium overnight. Cells were treated with or without NE (10 μM). After 48 hours incubation at 37 °C, cells were counted by Coulter particle Counter (Beckman coulter, Brea, CA).

### Immunofluorescence staining

Immunofluorescence staining were performed according to previously described protocols^49^. Human dermal fibroblasts were treated with or without NE (10 μM) for 1 hour, and then fixed in 4% paraformaldehyde (PFA) in PBS for 30 minutes. After being blocked with 3% drymilk–PBS supplemented with 5% normal goat serum for 1 hour, cells were stained with rabbit anti-α1AR antibody (Invitrogen) followed by Alexa 488–conjugated secondary Ab (Invitrogen). Cells were mounted in ProLong Gold antifade reagent (Invitrogen).

### Statistical analysis

*P* values were calculated by analysis of one-way ANOVA followed by Bonferroni’s post test. Error bars represent standard errors of the mean, and numbers of experiments (n) are as indicated.

## Additional Information

**How to cite this article**: Uehara, A. *et al*. Mechanistic insight into the norepinephrine-induced fibrosis in systemic sclerosis. *Sci. Rep.* **6**, 34012; doi: 10.1038/srep34012 (2016).

## Supplementary Material

Supplementary Information
