# Supplementary material for: Mechanistic insight into the norepinephrine-induced fibrosis in systemic sclerosis
Source: Sci Rep. 2016 Sep 21;6:34012. doi: 10.1038/srep34012 (PMC5030663; doi:10.1038/srep34012)
Supplement: Supplementary Information [file srep34012-s1.pdf]

Supplementary Data

Mechanistic insight into the norepinephrine-induced fibrosis in systemic sclerosis

Akihito Uehara[1], Sei-ichiro Motegi[1], Kazuya Yamada[1], Akihiko Uchiyama[1], Buddhini Perera[1], Sayaka Toki[1], Sachiko Ogino[1], Yoko Yokoyama[1], Yuko Takeuchi[1] & Osamu Ishikawa[1]

[1]Department of Dermatology, Gunma University Graduate School of Medicine, Japan

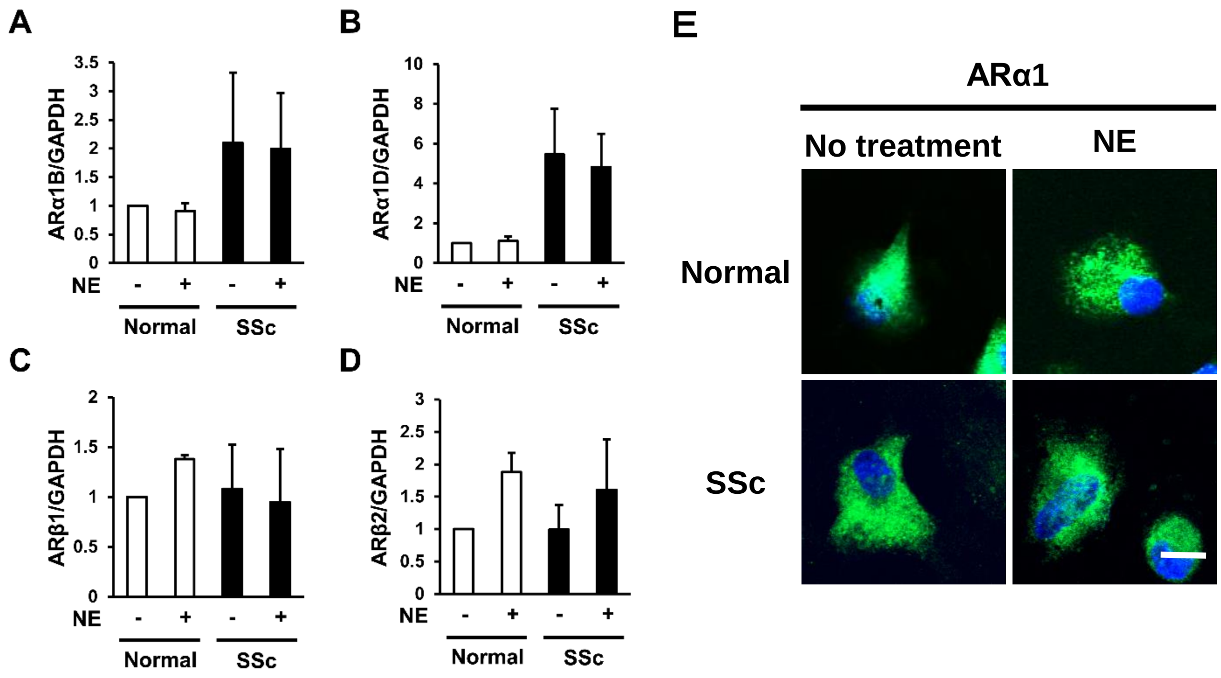

Figure S1. Expression of ARα and ARβ in normal and SSc fibroblasts. (A) Effects of NE on ARα1B mRNA in normal and SSc fibroblasts. **(B)** Effects of NE on ARα1D mRNA in normal and SSc fibroblasts. **(C)** Effects of NE on ARβ1 mRNA in normal and SSc fibroblasts. **(D)** Effects of NE on ARβ2 mRNA in normal and SSc fibroblasts. Normal and SSc fibroblasts were incubated for 1 hour with or without 10 μM NE. mRNA was measured using quantitative RT-PCR (n=3 samples). mRNA level in normal fibroblasts without NE was assigned a value of 1. All values represent mean ± SEM. (**E**) Effects of NE on the expression of ARα1 in normal and SSc fibroblasts by immunofluorescence staining. Scare bar = 20 μm.

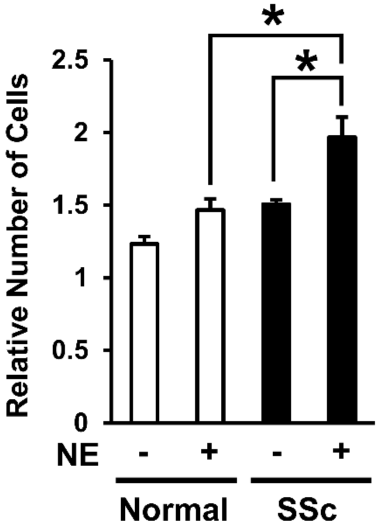

Figure S2. Proliferation of normal and SSc fibroblasts treated with or without NE.

Cell number of normal and SSc fibroblasts at the time when assay was started (5×10^5 cells) was assigned a value of 1. n=3 samples. All values represent mean ± SEM. *P<0.05.

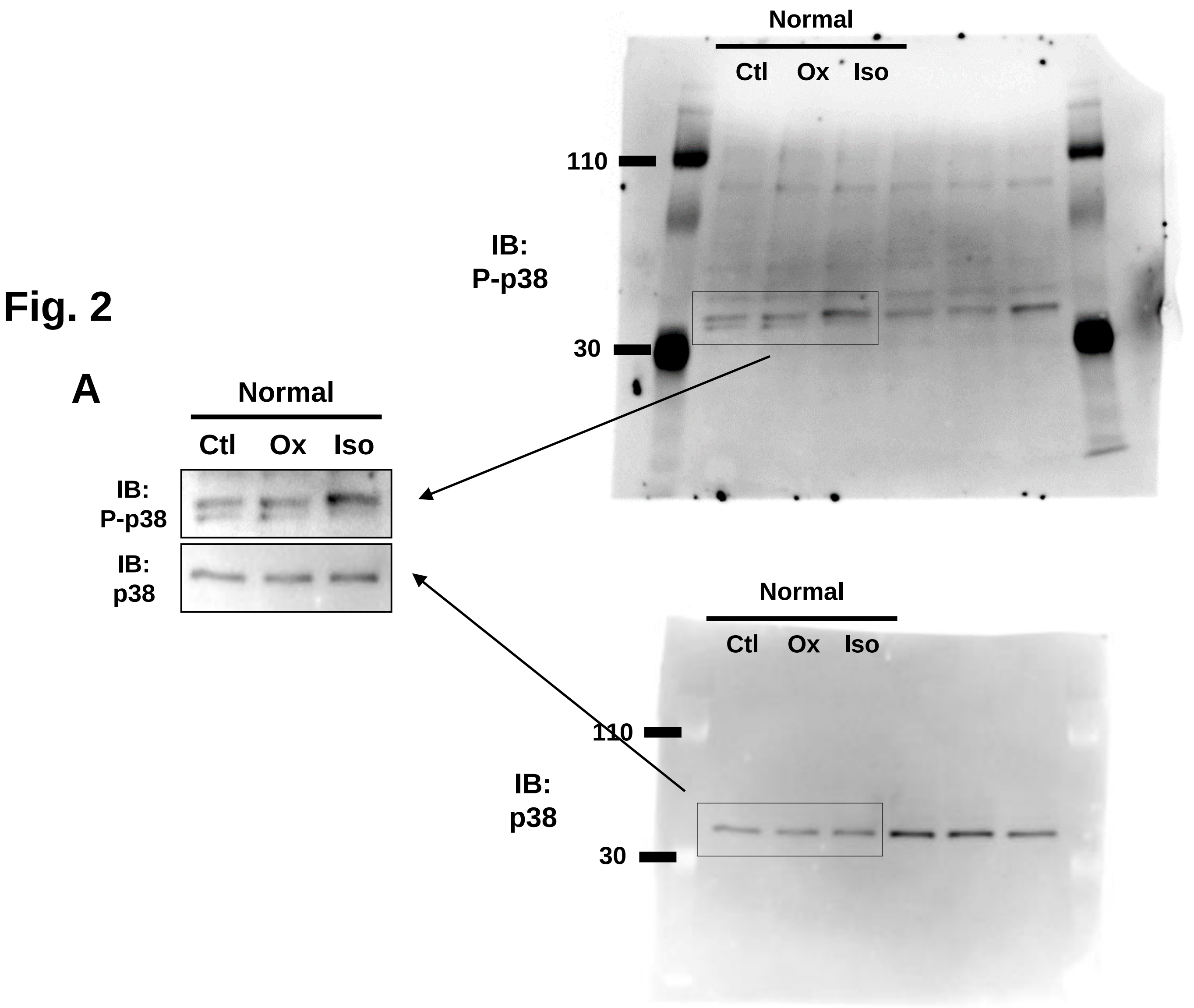

Figure S3. Full-length images of the immunoblots in Figure 2A.
Black line boxes indicate the cropped images used in Figure 2A.

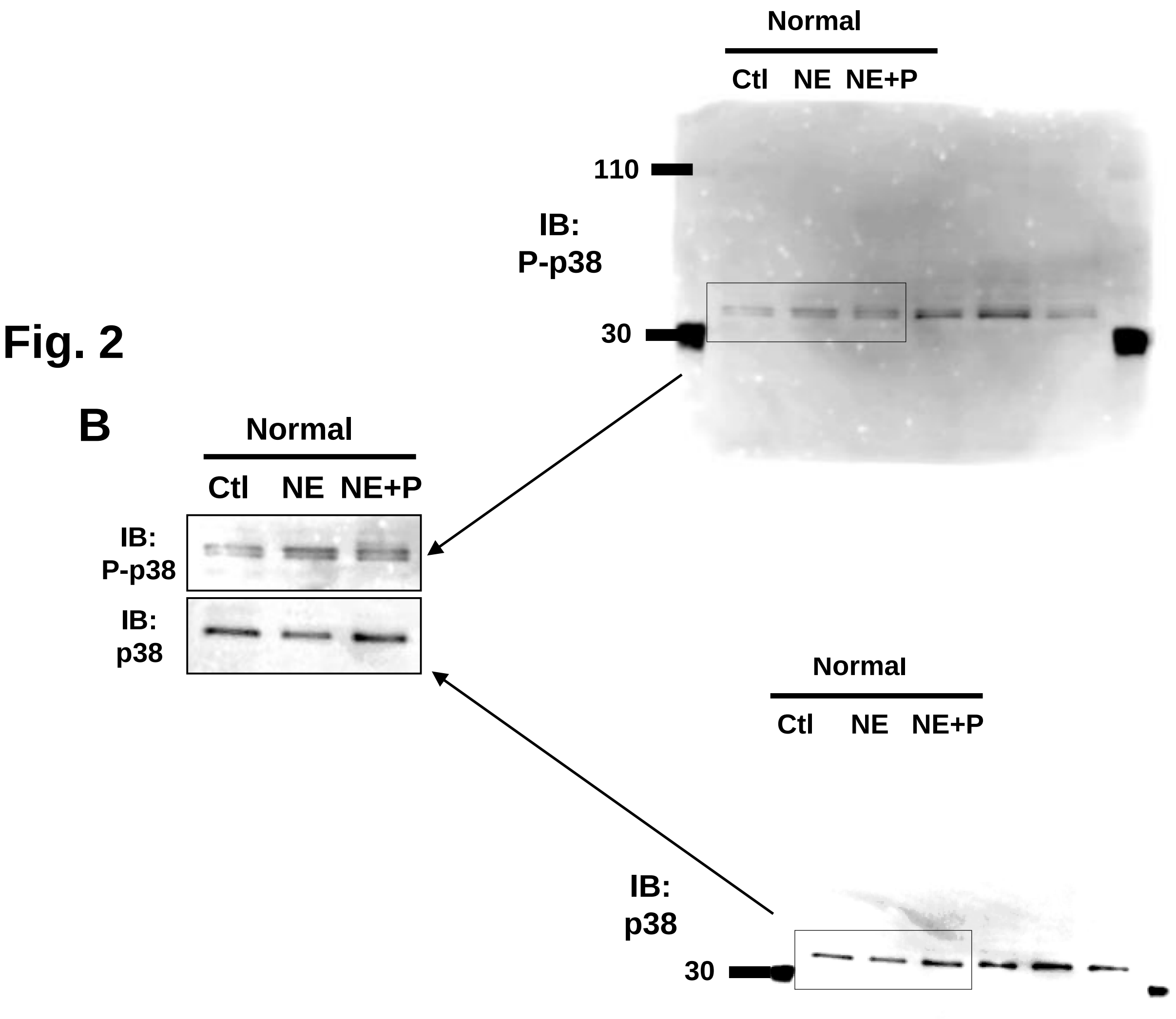

Figure S3. Full-length images of the immunoblots in Figure 2B. Black line boxes indicate the cropped images used in Figure 2B.

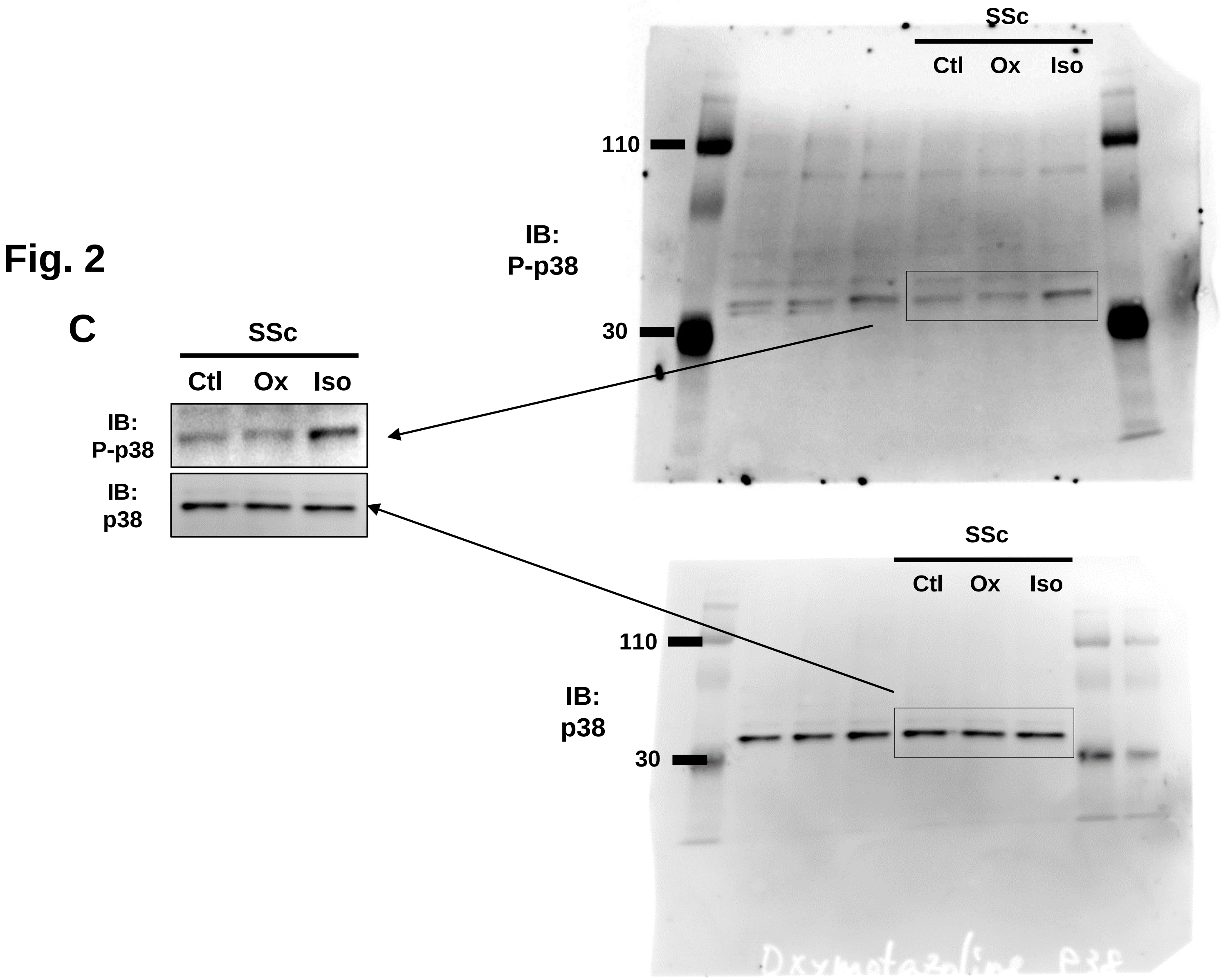

Figure S3. Full-length images of the immunoblots in Figure 2C. Black line boxes indicate the cropped images used in Figure 2C.

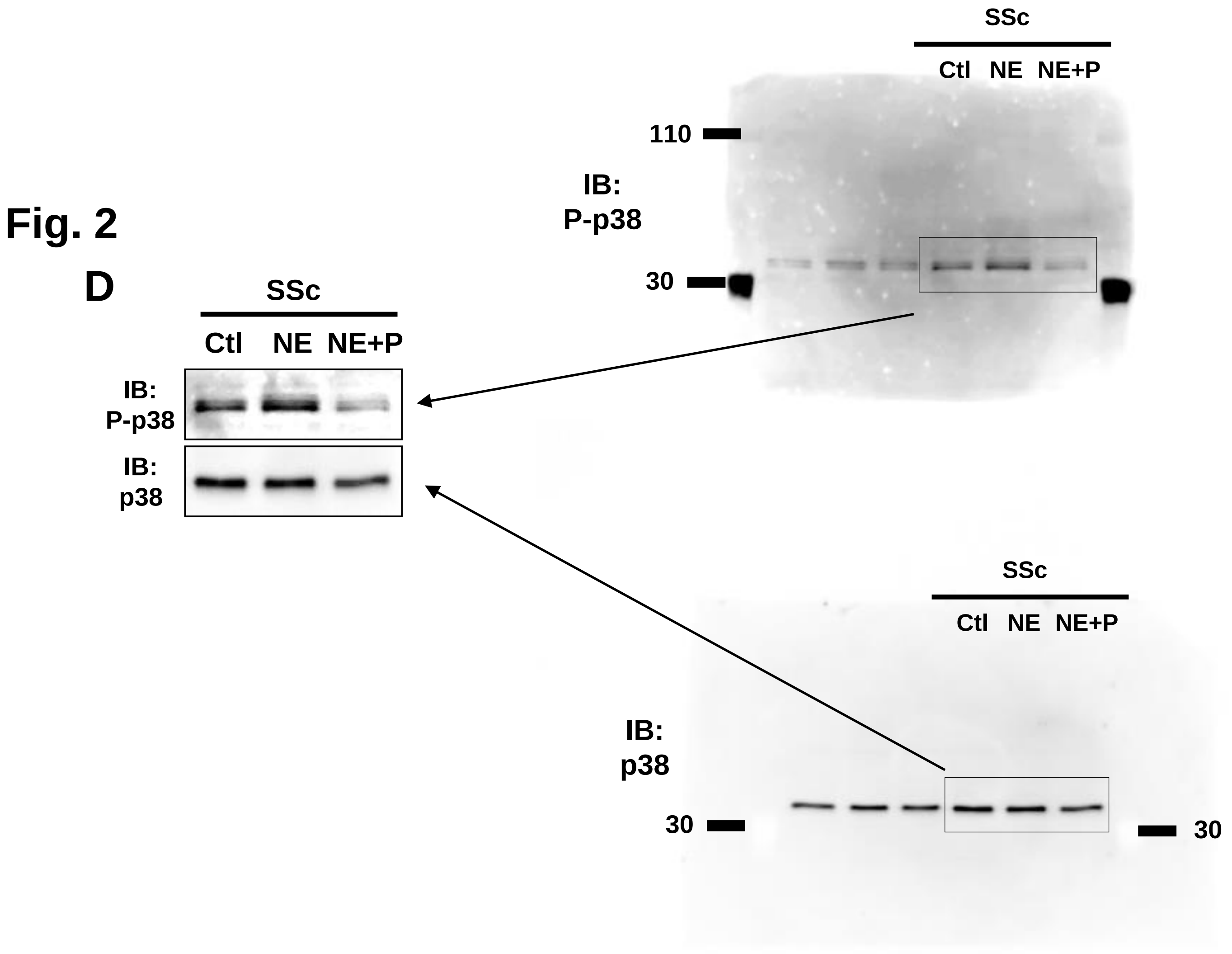

Figure S3. Full-length images of the immunoblots in Figure 2D.
Black line boxes indicate the cropped images used in Figure 2D.

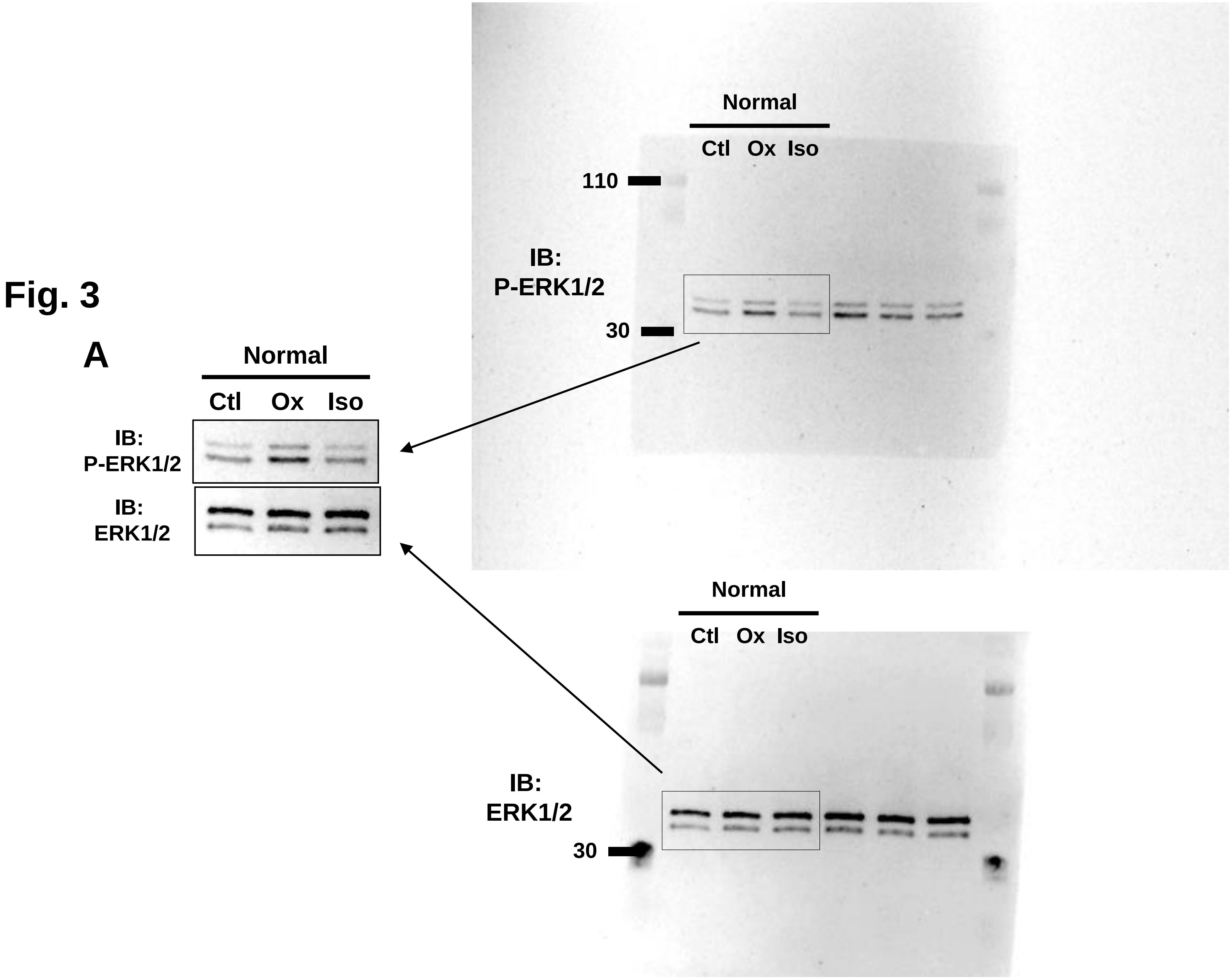

Figure S4. Full-length images of the immunoblots in Figure 3A. Black line boxes indicate the cropped images used in Figure 3A.

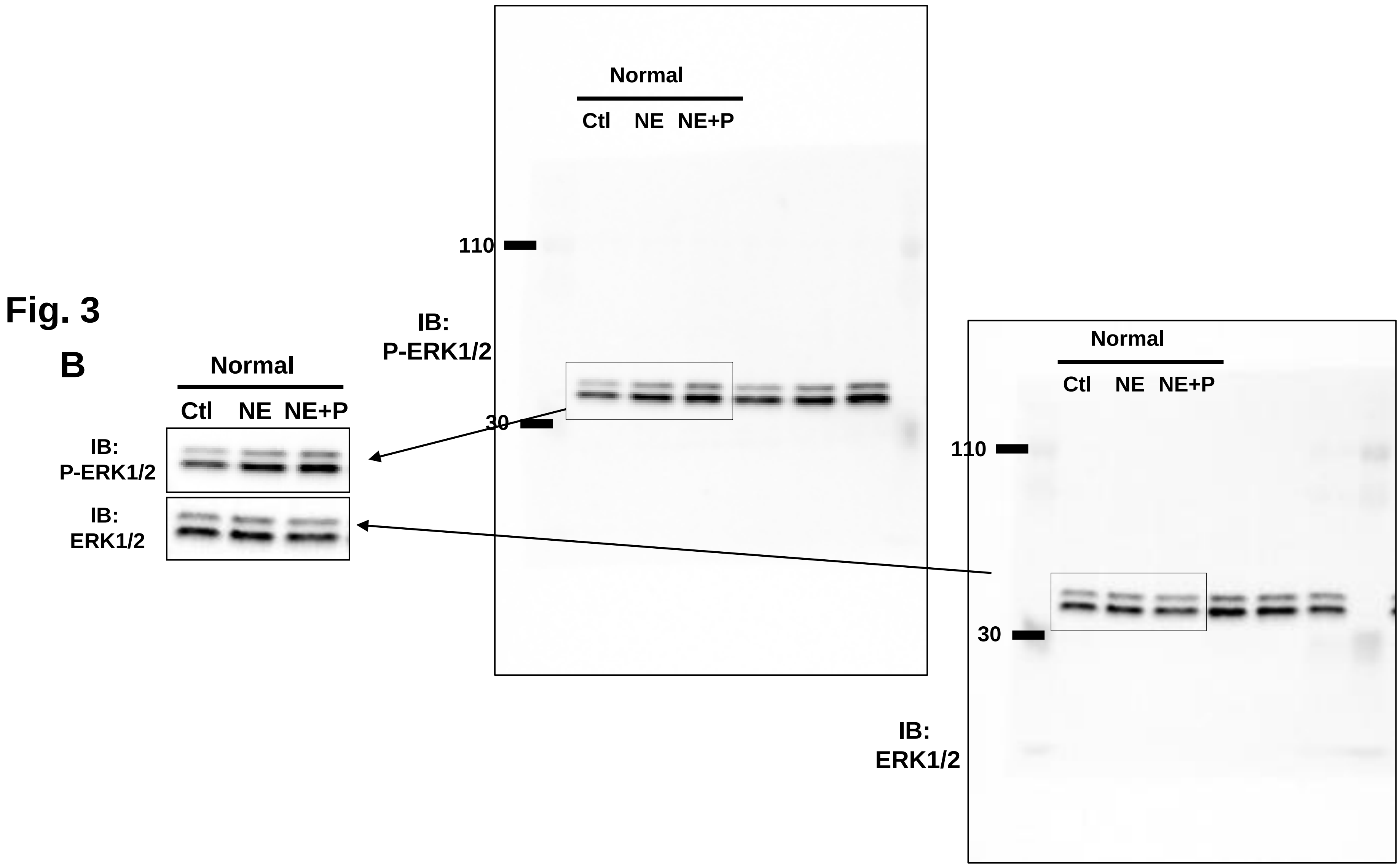

Figure S4. Full-length images of the immunoblots in Figure 3B.
Black line boxes indicate the cropped images used in Figure 3B.

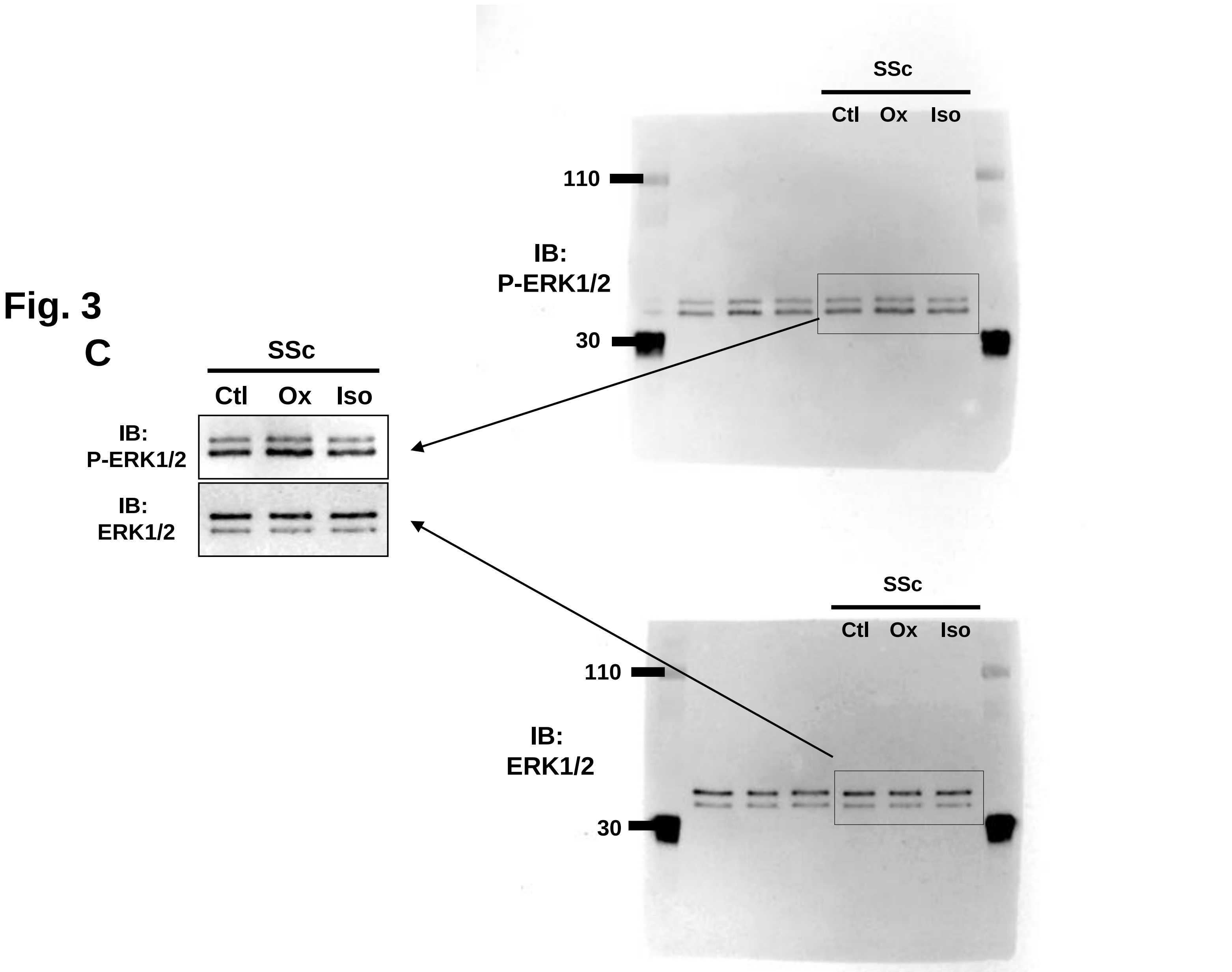

Figure S4. Full-length images of the immunoblots in Figure 3C.
Black line boxes indicate the cropped images used in Figure 3C.

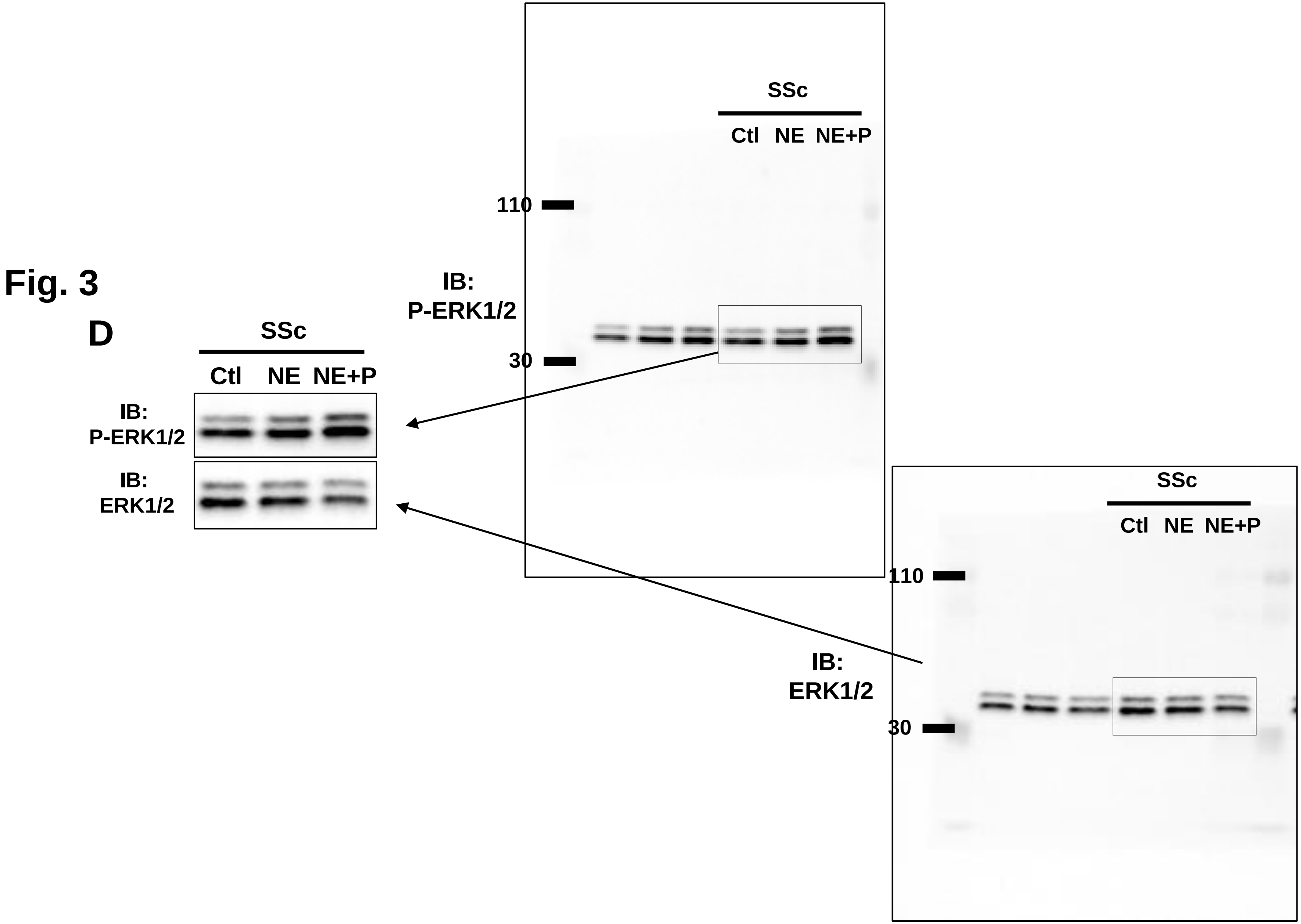

Figure S4. Full-length images of the immunoblots in Figure 3D.
Black line boxes indicate the cropped images used in Figure 3D.

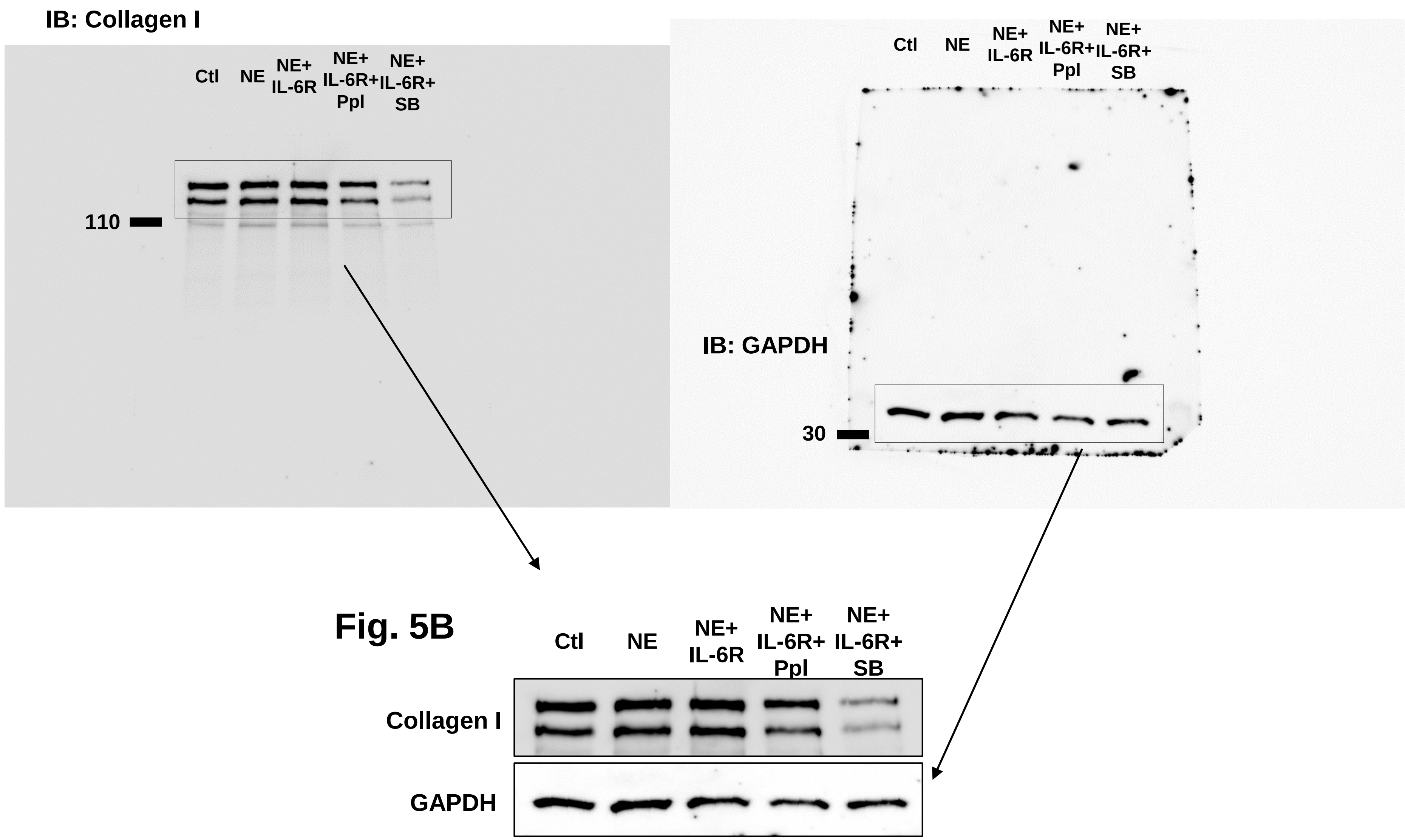

Figure S5. Full-length images of the immunoblots in Figure 5B.
Black line boxes indicate the cropped images used in Figure 5B.

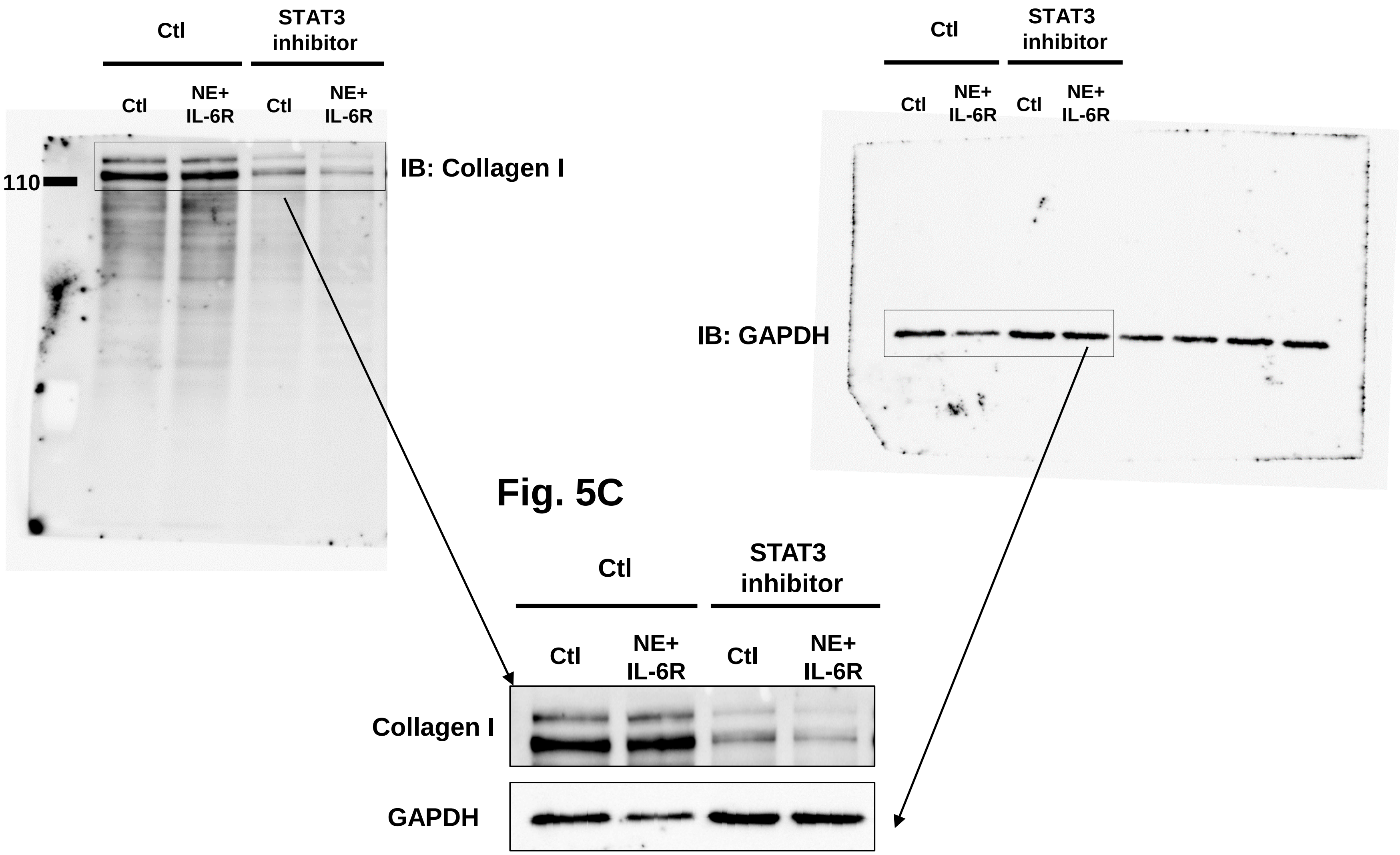

Figure S5. Full-length images of the immunoblots in Figure 5C.
Black line boxes indicate the cropped images used in Figure 5C.
